# Newer New Jersey Secondary School Teachers Study, 2021–2023: Insights Pertaining to Indoor Air Quality and Safety

**DOI:** 10.3390/ijerph23030371

**Published:** 2026-03-14

**Authors:** Derek G. Shendell, Juhi Aggarwal, Midhat Rehman, Maryanne L. Campbell

**Affiliations:** 1 New Jersey Safe Schools Program (NJSS), Rutgers School of Public Health (SPH), Rutgers University, 683 Hoes Lane West, 3rd Floor SPH—Suite 399, Piscataway, NJ 08854, USA; ja880@sph.rutgers.edu (J.A.); mr1638@sph.rutgers.edu (M.R.); mlf159@sph.rutgers.edu (M.L.C.); 2 Department of Environmental & Occupational Health & Justice, Rutgers School of Public Health (SPH), Piscataway, NJ 08854, USA; 3 Environmental & Occupational Health Sciences Institute, Rutgers University, Piscataway, NJ 08854, USA

**Keywords:** cleaning, sanitizing, and disinfecting products, indoor air and environmental quality in schools, school buildings, school teacher safety and health, ventilation

## Abstract

**Highlights:**

**Public health relevance—How does this work relate to a public health issue?**

- People spend most of their time indoors, and for teachers, this includes time at work, such as in schools, and at home, plus some time spent in transit (bus, car, train, etc.).
- Indoor air and environmental quality are critical concerns for school safety and health, with increased importance after the COVID-19 pandemic.

**Public health significance—Why is this work of significance to public health?**

- To date, few data exist on K-12 teacher-specific opinions and concerns about safety, health, and non-gun violence-related environmental topics.
- This study provides data on awareness, attitudes, concerns, and perceptions of newer New Jersey public secondary school teachers to inform evidence-based recommendations for policy and practice in schools.

**Public health implications—What are the key implications or messages for practitioners, policy makers, and/or researchers in public health?**

- To help alleviate teacher concerns with using cleaning and disinfecting products indoors, during and outside normal school hours, install more mechanical ventilation systems or portable air cleaners, and focus on providing products with less harmful chemical ingredients.
- When outdoor air quality conditions and weather factors like humidity, temperature, and wind are favorable, windows could be retrofitted to be potentially operable for some degree of opening to provide natural ventilation, if available.

**Abstract:**

Few studies focus on levels of concern among teachers regarding safety and health (S&H) such as indoor air quality and related environmental S&H topics in K-12 schools. Between October 2021 and June 2023, the New Jersey (NJ) Safe Schools Program provided work-based learning training to 163 newer NJ public secondary career and technical education teachers and asked them to complete online surveys regarding school S&H during the COVID-19 pandemic. There were 205 total survey entries out of 436 possible entries from multiple surveys (two surveys plus a follow-up survey in fall 2022 for those trained in 2021-22 SY). This paper focuses on concerns and perceptions of teacher S&H in physical workplaces with or without ventilation; perceived safety of cleaning, sanitizing, and disinfecting products (CSDPs); and who is responsible for school S&H. About half of the participants were “very concerned/concerned” about the health effects of CSDPs, and most believed principals are responsible for school S&H. School administrators and principals should take teacher concerns into account to develop, with safety professionals, relevant procedures, including for CSDP use, and provide adequate mechanical ventilation in classrooms.

## 1. Introduction and Background Context

School safety is a complex subject and includes the range of exposure agents or hazards to human health: biological, chemical, physical (ergonomics), radiological, and social or psychosocial stressors. The physical and mental safety, health and wellness (S&H) of teachers and educational support professionals are crucial to cultivating healthy school environments [1,2,3,4,5]. The Whole School, Whole Community, Whole Child model by the U.S. Centers for Disease Control and Prevention focuses on ten components; the two related to teachers are physical environment and employee wellness [5]. The physical environment includes the school’s indoor air and environmental quality plus its physical conditions (e.g., mechanical and/or natural ventilation, moisture, temperature, noise, and lighting) along with protection from physical (e.g., violence including crime and injury), biological (e.g., mold), and chemical threats (e.g., cleaning and disinfecting agents). Employee wellness considers both mental health and physical health, including ergonomics. Strategies promoting healthy school environments include inspecting and maintaining facilities and spaces [6], involving facilities managers/directors and custodians, plus safety professionals working for K-12 districts or specific schools.

Indoor air and environmental quality re-emerged as a critical concern for school S&H due to the COVID-19 pandemic. School materials such as chalk, scented markers, and cleaning, sanitizing, and disinfection products (CSDPs) may introduce additional pollutants [7,8,9,10]. In a recent study, most New Jersey (NJ) career and technical education (CTE) teachers reported buying CSDPs for use in their classrooms with only a third of them reading the labels, as they have been trained to do based on the Hazard Communication Standard of the U.S. Department of Labor-Occupational Safety and Health Administration, 29 CFR 1910.1200 (adopted by reference in NJ), to check if they are marketed as ecofriendly [8].

Schools often have a higher number of people in each area compared to typical office spaces. Effective ventilation and filtration strategies, with mechanical systems, air cleaners, and fans, plus natural ventilation via operable doors and windows as safely possible, can protect, promote, and enhance S&H in K-12 schools [11,12,13,14].

To date, there are few data regarding K-12 teacher-specific opinions on how concerned they are regarding specific S&H topics and non-gun violence related environmental matters in schools [15,16,17,18,19]. Teachers, like adult workers in general, are exposed to a range of workplace hazards and may potentially lack initial and ongoing training [18,19]. For example, in 2019, about 19.0% of Australian teachers did not feel safe at schools; about 54.0% of the respondents who provided further comments mentioned violence, aggression, and/or physical assault as the reason they feel unsafe [18]. More recently, another example of how and why teachers felt unsafe in schools during the COVID-19 pandemic after returning to in-person education was the fear of contracting COVID-19 while instructing students or working in classrooms [19].

Therefore, the NJ Safe Schools Program (NJSS) asked newer NJ secondary school teachers who took the work-based learning (WBL) supervisory courses to answer anonymous online S&H surveys [8,13,20,21,22]. Specifically, in this brief, we assessed questions on general perceptions and concerns about both S&H in school physical environments and about CSDP use [23,24], including who was responsible for school S&H during school years impacted by the COVID-19 pandemic. These new data from secondary school teachers trained in areas like business, including math, marketing, sciences, engineering, and technologies, including graphic design, architecture, and construction who are new to CTE WBL programs [25,26] can help inform general school S&H plans as well as emergency preparedness and response procedures for future epidemics/pandemics and natural disasters/extreme weather events.

## 2. Materials and Methods in Brief

In the 2021–2022 and 2022–2023 public school years (SY), NJSS provided WBL training to 163 newer NJ CTE teachers. The survey questions were adapted or used directly as previously validated questions from other federal agencies and prior NJSS research surveys to target topics known to be important to CTE teachers [8,13,20,21]. For other, more specific details of the inclusion and exclusion criteria, along with more information on the trainings, data collection, and data analysis procedures, and justifications for stratification beyond those briefly summarized below, please see other papers from the overall study [8,13,20,21,22]. While the overall study’s surveys focused on several topics regarding the physical school environment, attitudes towards CSDPs, S&H of employees including physical and mental health during COVID-19, etc., this paper focused explicitly on how safe these newer NJ teachers feel at their secondary schools, whether these newer NJ teachers feel they have concerns about being/feeling safe in schools, and thus who is responsible in their opinion for S&H in schools. These exact questions appear as bullet points below. Table 1 with selected results by age group includes the exact wording of some questions.

- Overall, how safe do you feel in your school workplace? How concerned are you about your health and safety at your school campus?
- How concerned are you about your health and safety at your assigned school classroom/office space?
- At school, who is responsible for S&H?

Across three surveys, 205 of a possible 436 completed or partially completed entries were received. (Note: 163 participants got both surveys 1–2, and 110 participants got the follow-up survey since they completed training before summer 2022). Of those who answered questions, 41.9% identified as males and 59.1% identified as non-Hispanic White. Most (65.2%) teachers had a master’s degree or doctoral degree, with an average of about six years of post-secondary education. About half (51.8%) of teachers were from North NJ, and 24.1% of teachers were from Central NJ and South NJ. The teachers had worked an average of about 13 years total (in CTE and non-CTE) at the time of the surveys. Further demographic details can be found in other papers from the overall study [8,13]. Data were used to determine differences between different counties of work, year of training, age, gender identity, and race/ethnicity.

The term “safe” was not defined for participants; therefore, participants answered survey questions based on their subjective personal definition and perception of feeling “safe.”

Finally, after data management and initial descriptive statistics were computed for the entire study population, this paper’s data were stratified by county of work, the year the training was taken, age, gender identity, and race/ethnicity. Fisher’s Exact tests were used to compare the distinct groups. This test was used due to small sample sizes of values, and missing data were excluded from the analysis. In this study, *p*-values below 0.05 were considered statistically significant. Data were managed using Microsoft Excel and analyzed using SAS analytics software 9.4 (Cary, NC, USA).

The Institutional Review Board (IRB) at Rutgers, the State University of New Jersey, granted approval for this study.

## 3. Results

Most (94.5%) teachers felt very or moderately safe in schools. There were no differences between stratifications except for age, where those who were younger (22–41-year-olds) were less likely to generally feel very safe at school when compared to older groups (42+) (25.7% 22–41 vs. 50.9% 42+, *p* = 0.05 (Table 1).

About one-third of participants were “very concerned/concerned” about S&H on their school campus, in the classroom, and in the office space. Males were more likely to feel “very concerned/concerned” towards S&H in their classroom compared to females (47.4% male vs. 24.0% female, *p* = 0.003).

A majority (90%) of participants considered concerns for S&H in schools important or very important. Almost half (46.5%) were “very concerned/concerned,” and 18.8% were unconcerned about ingredients in CSDPs, with no differences across stratifications. The S&H concerns included volatile organic compounds (VOCs) released from CSDPs may contribute to poor indoor air quality [23]. This is a well-known exposure; VOCs may cause skin irritation and respiratory issues, among other adverse health effects for adults and children [24].

Teachers spend time in sub-environments (classrooms, eating areas, play/exercise areas, and outdoor recreational areas). Most teachers (95%) want to be healthy and feel safe at schools, as noted above, and then when asked who was responsible for S&H in schools, overall, 71.7% of participating teachers thought the school’s principal was responsible. Additionally, a further 47.8% believed it was other staff, 43.5 believed it was the facilities or operations and maintenance staff, and 38.0% believed it was the teachers. Since this was a “select all that apply” question, it must be noted that 21% of participants chose every answer option provided. As a result, it can be noted how younger participants, as compared to older participants, were more likely to believe both the facilities or operations and maintenance staff (60.0% under 42 vs. 33.3% 42 and over, *p* = 0.02) as well as the teachers (60.0% under 42 vs. 24.6% 42 and over, *p* ≤ 0.001) have responsibility for school S&H (Table 1).

## 4. Discussion

This study contributes to ongoing conversations about how individual characteristics may shape perceptions of S&H and equity in educational environments, and highlights different concerns among different demographic groups, suggesting the need for various interventions. Implementing an integrated design approach to feature improved indoor air and environmental quality via both visual comfort (lighting) and thermal comfort (with consistent indoor temperatures) may improve teacher satisfaction and retention [11,27,28,29,30,31]. Teachers demonstrated concern about indoor air quality via CSDP use in their classroom workspaces and the chemical ingredients of these products when released into the air [7,8,9,10]. By prioritizing indoor air and environmental quality and putting mechanical ventilation systems in classrooms, teachers may be less concerned about CSDPs.

### Strengths and Limitations

The strengths of the study were the survey was distributed online, which allowed data to be collected, stored, managed, and analyzed digitally, and teachers completed the surveys at their own pace with response anonymity, which also allowed teachers to be open about their opinions with no fear of repercussions for honesty. However, anonymity can also be considered a limitation, as we cannot determine if multiple people took surveys from the same school computers available to teachers, or if a few participants possibly took any of the surveys multiple times. This specific caveat was further discussed in other papers from our overall study [8,13,20,21]. Furthermore, it should be reiterated this study was not a probability sample and not representative of all NJ secondary school teachers, though possibly the data are representative of newer NJ secondary school teachers who completed the required training to supervise students in CTE WBL or school-sponsored structured learning experiences in approved programs of study.

Since this study did not define the term “safe,” participants answered survey questions based on their personal definition of feeling “safe.” Additionally, feelings of safety were not assessed for each survey topic, but rather an overall assessment of general safety. 

Another known pair of limitations was related to the specific study population. These were newer public NJ secondary school CTE teachers [25,26], with their relatively small sample size of 205 participants across two SY. NJ teachers in secondary school career-technical education work daily in classrooms, shops, laboratories, etc., on-campus and supervise students off-campus for their WBL or structured learning experiences in approved NJ programs of study. Other details of these indoor (and sometimes semi-enclosed or outdoor) micro-environments used were not ascertained. For example, we did not quantitatively measure anything in the field in this study, and we did not ask about operable windows, though it should be noted how in NJ—and in many other U.S. states and countries—teachers use classrooms, laboratories, shops, etc., without windows and/or doors to the outdoors/exterior of buildings. Future research should strive to ascertain greater individual-level teacher workplace details for the study population, given when outdoor air quality conditions and weather factors like humidity, temperature, and wind are favorable and if windows are present or could be made available during modernization activities, then those windows could be retrofitted to be potentially operable for some degree of opening to provide supplemental natural ventilation.

## 5. Conclusions and Recommendations

In conclusion, this study during school years impacted by the COVID-19 pandemic provided new data on perspectives of teachers new to career-technical education and suggested most public secondary or high school teachers are concerned about S&H in schools, in general and in assigned classrooms, shops, and/or laboratories, and how younger teachers tend to feel less generally safe in schools when compared to older teachers.

The practical implications suggest two recommendations for policy and practice in K-12 schools:

1. To help alleviate teacher concerns with using chemical CSDPs indoors, during and outside normal school hours, install more mechanical ventilation systems or portable air cleaners, and focus on providing products with less harmful chemical ingredients.
2. K-12 school districts and their facilities/operations and maintenance staff, plus the contracted consulting safety professionals and industrial hygienists, can further develop policies and practices—both routine and for emergency preparedness and response—to help teachers and other education professionals feel safe in schools and address reported real and perceived S&H concerns from consumer product use. Furthermore, there should be clear guidelines and regular meetings regarding general S&H in schools, and at these meetings, identify and discuss who is responsible for different aspects of safety protocols and procedures in school buildings.

## Figures and Tables

**Table 1:** Participating teacher perceptions of responsibility for school safety and health (S&H) by age.

Question, and Answer Options	Ages 22–41 (n = 35)	% of Total	Ages 42+ (n = 57)	% of Total	Fisher’s Exact Test	Total ^a^ (n = 92)	% of Total ^b,c^
Overall, how safe do you feel in your school workplace?					0.05 *		
Very Safe	9	25.7%	29	50.9%		38	41.3%
Moderately Safe	24	68.6%	25	43.9%		49	53.3%
Not Really Safe	2	5.7%	3	5.3%		5	5.4%
At school, who is responsible for S&H? (pick all that apply):							
Facilities or operations and maintenance staff	21	60.0%	19	33.3%	0.02 *	40	43.5%
Principal	28	80.0%	38	66.7%	0.23	66	71.7%
Teacher	21	60.0%	14	24.6%	<0.001 **	35	38.0%
Other staff	20	57.1%	24	42.1%	0.20	44	47.8%

^a^ Includes questions/participants from survey 1 and follow-up survey. ^b^ This is a “choose all that apply” question, meaning total percentages will be over 100%. This implied participants viewed facilities/operations and maintenance staff, a school principal, and/or teachers as co-responsible. ^c^ No missing participants in this group. * *p*-value is less than 0.05, ** *p*-value is less than 0.001.

## Data Availability

The original contributions presented in this study are included in the article. Further inquiries can be directed to the corresponding author.

## Abbreviations

The following abbreviations are used in this manuscript:

CDC	U.S. Centers for Disease Control and Prevention
CSDPs	Cleaning, sanitizing, and disinfecting products
CTE	Career and technical education
IAQ/IEQ	Indoor air and environmental quality
NJSS	New Jersey Safe Schools Program
S&H	Safety and Health
SY	School Year
WBL	Work-based learning

## References

[1] American Federation of Teachers (2018). 2017 Educator Quality of Work Life Survey. https://www.aft.org/reports/2017-educator-quality-work-life-survey.

[2] Mehta S.B., Cornell D., Fan X., Gregory A. (2013). Bullying climate and school engagement in ninth-grade students. J. Sch. Health.

[3] Yablon Y.B., Addington L.A. (2017). Students’ feeling of safety in schools: Does frequency of victimization matter?. Am. J. Crim. Justice.

[4] Williams S., Schneider M., Wornell C., Langhinrichsen-Rohling J. (2018). Student’s Perceptions of School Safety: It Is Not Just About Being Bullied. J. Sch. Nurs..

[5] (2023). Centers of Disease Control and Prevention, Whole School, Whole Community, Whole Child (WSCC). https://www.cdc.gov/healthyschools/wscc/index.htm.

[6] (2021). Centers of Disease Control and Prevention, Components of the Whole School, Whole Community, Whole Child (WSCC)|Whole School, Whole Community, Whole Child (WSCC). https://www.cdc.gov/healthyschools/wscc/components.htm.

[7] (2023). American Lung Association, from Absences to Aces: Transforming Education with Healthy Classroom Air. https://www.lung.org/blog/healthy-classroom-air-steps.

[8] Aggarwal J., Campbell M.L., Rehman M., Nguyen K.T., Shendell D.G. (2024). Perspectives and Attitudes of Newer New Jersey High School Teachers towards Cleaning, Sanitizing, and Disinfecting Consumer Products Used in School Classrooms. Int. J. Environ. Res. Public Health.

[9] Jovanovic M., Vucicevic B., Turanjanin V., Zivkovic M., Spasojević V. (2014). Investigation of indoor and outdoor air quality of the classrooms at a school in Serbia. Energy.

[10] United States Environmental Protective Agency (2013). Safer Chemical Ingredients List [Data and Tools]. https://www.epa.gov/saferchoice/safer-ingredients.

[11] (2023). United States Environmental Protection Agency Indoor Air Quality in High Performance Schools|US EPA. https://www.epa.gov/iaq-schools/indoor-air-quality-high-performance-schools.

[12] Shaughnessy R.J., Haverinen-Shaughnessy U., Nevalainen A., Moschandreas D. (2006). A preliminary study on the association between ventilation rates in classrooms and student performance. Indoor Air.

[13] Aggarwal J., Shendell D.G., Nguyen K.T., Rehman M., Campbell M.L. (2024). Newer New Jersey Work-Based Learning Teachers During the COVID-19 Pandemic: School Safety Regarding Ventilation, Trainings, and Awareness of Government Agencies Resources. Int. J. Environ. Health Res..

[14] Allen J.G., Ibrahim A.M. (2021). Indoor Air Changes and Potential Implications for SARS-CoV-2 Transmission. JAMA.

[15] Aggarwal J., Eitland E.S., Fakeh Campbell M.L., Gonzalez L.N., Greenberg P., Kaplun E., Sahili S., Koshy K., Rajan S., Shendell D.G. (2021). Built/Physical Environment Attributes and Preparedness for Potential Gun Violence at Secondary Schools. J. Environ. Health.

[16] Booren L.M., Handy D.J., Power T.G. (2011). Examining Perceptions of School Safety Strategies, School Climate, and Violence. Youth Violence Juv. Justice.

[17] Bora H.T., Seheryeli M.Y., Altun S.A. (2023). Relationship between students’ sense of school belonging with principals’ perceptions of school discipline, and teachers’ perceptions of school safety in TIMSS 2019. Curr. Psychol..

[18] Heffernan A., Longmuir F., Bright D., Kim M. (2019). Perceptions Of Teachers And Teaching in Australia, Monash University. https://researchmgt.monash.edu/ws/portalfiles/portal/302824782/301566106_oa.pdf.

[19] Quansah F., Frimpong J.B., Sambah F., Oduro P., Anin S.K., Srem-Sai M., Hagan J.E., Schack T. (2022). COVID-19 Pandemic and Teachers’ Classroom Safety Perception, Anxiety and Coping Strategies during Instructional Delivery. Healthcare.

[20] Campbell M.L., Aggarwal J., Nguyen K.T., Shendell D.G. (2024). Perceptions of New Jersey Teachers About Mental Health and School Services Offered During the COVID-19 Pandemic. Future.

[21] Campbell M.L., Shendell D.G. (2024). Assessing Indicators of Mental Health Distress Among New Jersey High School Teachers During the COVID-19 Pandemic. Med. Res. Arch. (Eur. Soc. Med.).

[22] Rehman M., Campbell M.L., Aggarwal J., Nguyen K.T., Shendell D.G. (2024). Changes in Personal Wellness Habits Among Newer New Jersey Teachers During COVID-19 Impacted School Years. J. Healthy Eat. Act. Living.

[23] Temkin A.M., Geller S.L., Swanson S.A., Leiba N.S., Naidenko O.V., Andrews D.Q. (2023). Volatile organic compounds emitted by conventional and “green” cleaning products in the U.S. market. Chemosphere.

[24] Koksoy Vayisoglu S., Oncu E. (2021). The use of cleaning products and its relationship with the increasing health risks during the COVID-19 pandemic. Int. J. Clin. Pract..

[25] New Jersey Department of Education (2025). Certification: Career and Technical Education Teachers. https://www.nj.gov/education/certification/cte/.

[26] New Jersey Department of Education (2025). Career Readiness: Work-Based Learning. https://www.nj.gov/education/cte/secondary/wbl/.

[27] Daisey J.M., Angell W.J., Apte M.G. (2003). Indoor air quality, ventilation and health symptoms in schools: An analysis of existing information. Indoor Air.

[28] Mendell M.J., Heath G.A. (2005). Do indoor pollutants and thermal conditions in schools influence student performance? A critical review of the literature. Indoor Air.

[29] Bluyssen P. (2016). Health, comfort and performance of children in classrooms—New directions for research. Indoor Built Environ..

[30] American Lung Association (2023). Importance and Impact of Air Quality in Schools. American Lung Association. https://www.lung.org/clean-air/indoor-air/building-type-air-resources/at-school/importance-of-air-quality-in-schools.

[31] Lazzara V., Stanisci I., Malizia V., Alfano P., La Grutta S. (2025). A scoping review of review on school indoor air quality and respiratory health in schoolchildren: Evidence available, key concepts and knowledge gaps. Indoor Built Environ..

